# Characterization of Vortex Vein Drainage System in Healthy Individuals Imaged by Ultra-Widefield Optical Coherence Tomography Angiography

**DOI:** 10.1167/tvst.13.9.19

**Published:** 2024-09-18

**Authors:** Zhonghua Luo, Yue Xu, Xiaomei Xiong, Shengsong Huang, Subinuer Alimu, Jinli Cui, Kun Xu, Ching-Kit Tsui, Shuxin Fan, Kaixuan Cui, Shanshan Yu, Xiaoling Liang

**Affiliations:** 1 State Key Laboratory of Ophthalmology, Zhongshan Ophthalmic Center, Sun Yat-Sen University, Guangdong Provincial Key Laboratory of Ophthalmology and Visual Science, Guangdong Provincial Clinical Research Center for Ocular Diseases, Guangzhou, China

**Keywords:** choroidal vortex vein drainage system, healthy eye, ultra-widefield (UWF) optical coherence tomography angiography (OCTA)

## Abstract

**Purpose:**

The purpose of this study was to investigate the choroidal characteristics of vortex vein (VV) drainage systems in healthy individuals using ultra-widefield optical coherence tomography angiography.

**Methods:**

The mean choroidal thickness (ChT) and choroidal vascularity index (CVI) of each VV quadrant (24 × 20 mm^2^ scan mode; superotemporal [ST], superonasal [SN], inferonasal [IN], and inferotemporal [IT] quadrants) were calculated. Furthermore, intervortex venous anastomosis (IVA) was classified into temporal, superior, inferior, and nasal types.

**Results:**

A total of 207 healthy eyes were analyzed to find that the ST quadrant had the thickest choroidal layer and highest CVI (all *P* < 0.05). Among the four VV drainage quadrants, the mean ChT and CVI decreased in the sequence of ST, SN, IT, and IN (all *P* < 0.05). Moreover, men had a higher CVI than women in all VV quadrants (all *P* < 0.05). IVA was observed in all VV quadrants of 91 eyes (43.96%), and in the macular region of 33 eyes (15.94%).

**Conclusions:**

The ST drainage system was identified as the preferred VV drainage route in healthy eyes. Among the four VV drainage quadrants, the drainage system adhered to the ST–SN–IT–IN order of descending perfusion. Furthermore, age- and sex-related differences were noted in the choroidal VV drainage systems of healthy eyes. Additionally, almost half of the healthy eyes had IVA in their choroidal vessel networks.

**Translational Relevance:**

The VV drainage system may be considered a novel imaging biomarker for ocular diseases.

## Introduction

The choroid is an organization of vascularized membranes located between the retinal pigmental epithelium–Bruch's membrane complex and the sclera layer that supplies oxygen and nutrition to the outer retina and plays an important role in ocular blood circulation.^1^^,^^2^ The choroid is drained by vortex veins (VV), which form four segmental VV drainage systems.^3^^–^^5^ Each independent VV drainage system has a well-defined boundary, called watershed zones (the horizontal watershed runs through the optic disc and the macular region, and the vertical watershed runs through the papillomacular region).^4^^,^^6^ Effectively, the choroidal VV drainage system is responsible for controlling choroidal venous outflow.

Previously, most of our knowledge of the VV drainage system in healthy eyes was drawn from indocyanine green angiography (ICGA). For instance, Mori et al.^6^ evaluated 36 healthy eyes using ICGA to find that half of the sample had an imbalanced VV drainage system characterized by a superotemporal (ST) or superonasal (SN) preferential drainage route. In contrast, Bacci et al.,^7^ who conducted ultra-widefield (UWF) ICGA of healthy volunteers, observed a balanced pattern in the VV drainage system of healthy eyes. Notably, intervortex venous anastomosis (IVA) has been defined as an identifiable direct connection between adjacent quadrants of VV ampullas.^8^ Recent advancements in UWF optical coherence tomography (OCT) and optical coherence tomography angiography (OCTA) have offered new avenues for the noninvasive, three-dimensional evaluation of the choroidal vessels. Until now, no large-scale study has examined the VV drainage system of healthy eyes. Moreover, its characterization has also remained unclear.

In recent years, the concept of pachychoroid spectrum disorders has emerged in the context of choroid-related diseases, which include central serous chorioretinopathy, and polypoidal choroidal vasculopathy, among others.^9^^–^^12^ Notably, pachychoroid spectrum disorders are characterized by attenuation of the choriocapillaris overlying dilated choroidal veins and choroidal vascular hyperpermeability.^13^ Although a few recent studies have paid attention to the role of the VV drainage system in pachychoroid spectrum disorders using UWF-OCTA, the relationship between the two remains poorly understood. This highlights the urgent need to attain a comprehensive understanding of the characterization of the VV drainage system in healthy eyes, which is essential for advancing existing knowledge on the pathophysiology of pachychoroid spectrum disorders and other choroid-related diseases.

In view of the above observations, we aimed to evaluate the characteristics of the VV drainage system in healthy eyes using UWF-OCTA. We documented the mean choroidal thickness (ChT) and choroidal vascularity index (CVI) in the ST, SN, inferonasal (IN), and inferotemporal (IT) drainage quadrants, and assessed the relationship among the mean ChT, CVI, and age, and axial length (AL). Additionally, we evaluated the location distribution and frequency of IVA in each drainage quadrant.

## Methods

### Study Design

This observational study was conducted on 207 healthy eyes of 207 healthy subjects at the Zhongshan Ophthalmic Center from January 2023 to August 2023. This research strictly adhered to the principles outlined in the Declaration of Helsinki and was approved by the medical ethics committee of Zhongshan Ophthalmic Center, Sun Yat-sen University, China (ID: 2023KYPJ100). Furthermore, all participants provided their written informed consent.

### Population

We gathered extensive information about each participant, including their gender, age, height, weight, blood pressure, and medical and personal histories. All volunteers received detailed ophthalmologic examinations, including best-corrected visual acuity (BCVA) testing, slit-lamp examination, intraocular pressure (IOP) measurement (Topcon CT-1, Tokyo, Japan), AL measurement (IOLMaster 700; Carl Zeiss Meditec AG, Oberkochen, Germany), dilated fundus photography (Canon CR-2, Tokyo, Japan), and OCT/OCTA. To maintain consistency, all assessments were conducted between 8:00 AM and 12:00 PM on the same day.

The inclusion criteria for the study were as follows: (1) 20 years ≤ age ≤ 80 years, (2) 21 mm ≤ AL ≤ 26.5 mm, (3) −6.00 diopters (D) ≤ spherical equivalent ≤ 3.00 D, (4) BCVA ≥ 20/25, (5) 10 mm Hg ≤ IOP ≤ 21 mm Hg, and (6) OCTA image quality score (0–10) ≥ 7. The exclusion criteria were: (1) eyes affected by glaucoma, uveitis, or retinal, choroidal, or optic nerve diseases; (2) history of ocular surgery or treatment; (3) diabetes, hypertension, nephropathy, and other systemic diseases affecting choroidal circulation; (4) pregnancy and lactation; (5) long-term use of corticosteroids or vasoactive medications; and (6) consumption of > 100 mL coffee or alcohol within the last 24 hours or of > 800 mL water 1 hour before examination.^14^ In case both eyes of a participant met the criteria, the right eye was selected for inclusion in the study.

### Imaging Acquisition

**Figure 1.**
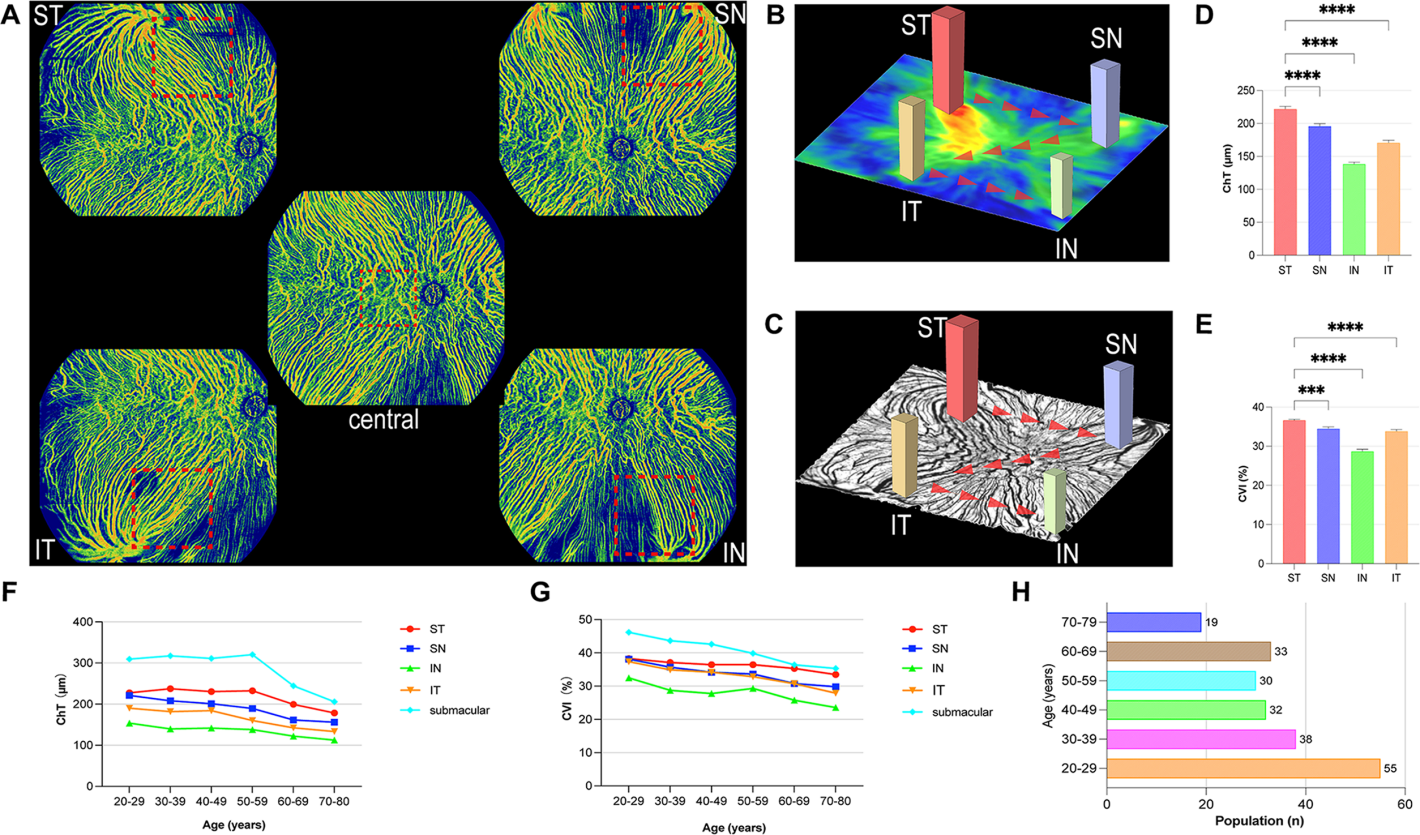
(**A**) OCTA imaging protocol. Five 24 × 20 mm^2^ choroid medium-to-large vessel images in predefined locations (central, ST, SN, IT, and IN quadrants) on the right eye of a healthy 50-year-old man were obtained. ChT and CVI were collected for a 9 × 9 mm^2^ area (*red dotted boxes*; ST, SN, IT, and IN quadrants) and a 6 × 6 mm^2^ area (*red dotted box*; central quadrant). The mean ChT (**B**) and CVI (**C**) decreased in the order of ST–SN–IT–IN. The ChT map (**B**) and choroid medium-to-large vessel image (**C**) were obtained from the right eye of a healthy 38-year-old man. (**D**) Bar graphs showing the quantification of the ChT of healthy eyes in the ST, SN, IN, and IT drainage quadrants. (**E**) Bar graphs showing the quantification of the CVI of healthy eyes in the ST, SN, IN, and IT drainage quadrants. Data are reported as mean ± SEM, based on repeated measures ANOVA. *P < 0.05, **P < 0.01, ***P < 0.001, and ****P < 0.0001. (**F**) Line graphs of ChT in the submacular area and the ST, SN, IN, and IT drainage quadrants for the 20 to 80 age group. (**G**) Line graphs of CVI in the submacular area and the ST, SN, IN, and IT drainage quadrants for the 20 to 80 age group. (**H**) Bar graphs of the population divided into age groups. OCTA, optical coherence tomography angiography; ST, superotemporal; SN, superonasal; IT, inferotemporal; IN, inferonasal; ChT, choroidal thickness; CVI, choroidal vascularity index; ANOVA, analysis of variance.

Choroidal imaging was conducted using a UWF swept-source OCTA device (BM-400K BMizar, TowardPi Medical Technology Co., Ltd., Beijing, China), which boasts a high scanning rate of 400,000 A-scans per second, a transverse resolution of 10 µm, and an optical in-depth resolution of 3.8 µm. Each participant underwent comprehensive 24 × 20 mm² OCTA scans across 5 predefined locations: the central, ST, SN, IT, and IN quadrants (Fig. 1A).^15^ All OCTA examinations were performed by an experienced technician (author X.L.).

To ensure data reliability, a stringent evaluation protocol was implemented. First, manual adjustments were made, if necessary, to ensure accuracy after automatic layer segmentation. Second, the quality of all images was assessed using built-in software (scoring ≥ 7 on a 0–10 scale) and then verified by two ophthalmologists (authors S.Y. and X.L.). Third, AL values were imported into the system to adjust for ocular magnification differences before imaging (Supplementary Fig. S1). Drawing on the Littmann formula (t = p × q × s; t = actual fundus dimensions, p = magnification factor of the imaging system, q = magnification factor for an individual eye, and s = value obtained from the imaging device) and the modified Bennett formula (q = 0.01306 × [AL - 1.82]), a new formula (t = [AL – 4] × s/20) was devised based on the optical design of the OCTA system and ray tracing techniques.^16^^–^^18^ This formula was implemented to correct the ocular magnification of the UWF-OCTA system. Notably, the BM-400K BMizar uses an aspherical lens with an 80-degree angle of view and an integrated optical path, which minimizes peripheral distortion, and also comprises a peripheral distortion correction algorithm. The consistency and quality of the OCTA images were scrutinized and validated by two experienced examiners (authors Z.L. and Y.X.), whereas a third professor (author X.L.) resolved any disagreements. Furthermore, to reinforce the reliability of the evaluation conducted in this study, intraclass correlation coefficients (ICCs) and Kappa coefficients (0.875) were used to assess examiner consistency (Supplementary Tables S1, S2).

### Three-Dimensional Analysis of the Choroidal Layer in the VV Quadrants and the Submacular Area

A vascular–stromal architecture was discernible in the OCT images, with vessels appearing as dark areas and stroma as light areas. This suggests that ChT and CVI (choroidal vascular volume/choroidal volume) can be considered potent biomarkers for examining choroidal circulation. Notably, ChT can be defined as the distance between Bruch's membrane (lower boundary of the retinal pigment epithelium) and the choroid-scleral interface, whereas CVI can be obtained from the medium-to-large vessel choroidal layer, which refers to the slab located between 29 µm beneath Bruch's membrane and the choroid-scleral interface. Built-in equipment with deep-learning algorithms was used for the automatic segmentation of the choroidal layer. Next, the ChT and CVI of the entire scanning area were automatically quantified by the deep learning algorithm in the OCTA device. To evaluate post-equatorial choroidal venous outflow, the mean ChT and CVI volumes pertaining to a 9 × 9 mm^2^ area were obtained, with the center of the largest VV ampulla being the vertex and the diagonal pointing toward the posterior fundus. The ends of each VV branch were connected to form a smooth curve, with the area enclosed within this curve defined as the area of the VV ampulla.^15^ Additionally, the choroidal layer in the submacular region was examined by conducting a 6 × 6 mm² OCT volumetric scan (see Fig. 1A).

### Classification Scheme For Intervortex Venous Anastomosis

Using Adobe Photoshop CS6 (Adobe Systems, Inc., San Jose, CA, USA), five 24 × 20 mm^2^ enface choroidal maps were combined with UWF choroidal topographic images centered on the macular region. The presence of IVA was assumed if two or more anastomotic vessels connected the VV ampullas in adjacent quadrants, with the connecting veins being greater than or equal to the size of the thickest retinal arcade vein located at the border of the optic disk.^19^ Notably, if the IVAs connected the ST and IT drainage systems, it was considered temporal IVA. Similarly, IVAs in the nasal (SN–IN), superior (ST–SN), and inferior (IT–IN) quadrants were classified. Along the same lines, macular IVA was deemed present if the anastomotic vessels passed through the macular region; otherwise, anastomosis was defined as peripheral IVA.

### Statistical Analysis

Statistical analyses were performed using the Statistical Program for Social Sciences 26.0 (IBM SPSS Inc., New York, NY, USA). To evaluate consistency in the measurement of choroidal parameters, ICCs were used between the two image examiners. The quantitative values were calculated as mean ± SD or mean ± SEM, whereas the categorical values were expressed as numbers (percentages). The normality of all continuous data was assessed using the Shapiro–Wilk test, whereas the equality of variances was confirmed using the Levene test. Furthermore, a Mann–Whitney *U* test was conducted to compare the continuous variables and categorical variables of the independent groups. In addition, repeated measures of analysis of variance (ANOVA) were obtained to identify the differences within groups. Additionally, Pearson's correlation analysis was performed to determine the associations of ChT and CVI with age and AL, which were graded as follows: 0 to 0.19 = very weak; 0.20 to 0.39 = weak; 0.40 to 0.59 = moderate; 0.60 to 0.79 = strong; and 0.80 to 1.00 = very strong. Notably, a *P* value of less than 0.05 was considered statistically significant.

## Results

### Participant Demographics

A total of 230 healthy eyes of the 230 participants who met the above-mentioned criteria were included in the analysis of the characteristics of the choroidal VV drainage system in normal Chinese eyes using UWF-OCTA (CCVC-OCTA). However, 23 participants were excluded for the following reasons: 11 cases presented poor quality images, 7 cases exhibited unclear choroidal layer boundaries, and 5 cases involved uncooperative examinations. Ultimately, 207 eyes of 207 Chinese participants were selected for further analysis. The demographics and clinical characteristics of these 207 participants are presented in Table 1. The mean age of the participants was 45.42 ± 16.92 years (range = 20.93–79.49 years). Furthermore, 117 participants (56.5%) were women, 18 (8.7%) were current smokers, and 35 (16.9%) were current drinkers. The mean AL of the selected eyes was 24.04 ± 1.13 mm (range = 21.41–26.49 mm), whereas the mean IOP was 14.33 ± 2.53 mm Hg (range = 10–21 mm Hg).

**Table 1.** Demographics and Clinical Characteristics

Characteristics	Total	Range
Eyes, *n*	207	N/A
Female, *n* (%)	117 (56.52)	N/A
Age, y	45.42 ± 16.92	20.93 to 79.49
Systolic pressure, mm Hg	121.56 ± 16.46	90 to 139
Diastolic pressure, mm Hg	78.96 ± 9.20	60 to 88
Height, cm	164.36 ± 8.28	146.50 to 193
Weight, kg	61.09 ± 10.70	34.45 to 110.80
Body mass index	22.54 ± 3.00	12.04 to 33.95
Axial length, mm	24.04 ± 1.13	21.41 to 26.49
Spherical equivalent, D	−3.34 ± 1.27	−5.85 to 0.50
Intraocular pressure, mm Hg	14.33 ± 2.53	10.00 to 21.00
Current smokers, *n* (%)	18 (8.70)	N/A
Current drinkers, *n* (%)	35 (16.91)	N/A

N/A, not available.

Values are presented as number (%) or mean ± SD.

### Differences Noted in the Choroidal Layer in the VV Quadrants and in the Submacular Area

First, we evaluated the UWF choroidal VV drainage system in two and three dimensions using UWF-OCTA technology (Table 2). The mean ChT of the VV in the ST, SN, IN, and IT drainage quadrants were 221.01 ± 55.69 µm, 195.98 ± 51.99 µm, 138.49 ± 40.08 µm, and 170.79 ± 52.01 µm, respectively. The mean CVI was 36.63% ± 3.81%, 34.51% ± 6.27%, 28.73% ± 7.21%, and 33.85% ± 6.00%, respectively. Notably, men demonstrated a higher CVI than women across all drainage quadrants, showing a statistically significant difference (ST = 37.33% ± 3.74% vs. 36.10% ± 3.80%, *P* = 0.023; SN = 35.42% ± 6.02% vs. 33.81% ± 6.39%, *P* = 0.023; IN = 30.02% ± 6.02% vs. 27.74% ± 7.46%, *P* = 0.001; and IT = 35.01% ± 6.29% vs. 32.97% ± 6.39%, *P* < 0.001). The ST quadrant exhibited the thickest choroid layer and highest CVI among all quadrants, suggesting a trend of ST > SN > IT > IN for both ChT and CVI (Figs. 1B–E, all *P* < 0.05).

**Table 2.** Demographic and Choroidal Characteristics of the Recruited Healthy Participants

Characteristics	Total	Male	Female	*P* Value^a^
Eyes, *n*	207	90	117	N/A
Age, y	45.42 ± 16.92	43.95 ± 17.67	46.55 ± 16.30	0.206^c^
Axial length, mm	24.04 ± 1.13	24.24 ± 1.09	23.88 ± 1.15	0.079^c^
Vortex vein's ChT, µm				
Superotemporal quadrant	222.01 ± 55.69	225.81 ± 59.75	219.09 ± 52.42	0.483^c^
Superonasal quadrant	195.98 ± 51.99	201.43 ± 56.33	191.79 ± 48.22	0.385^c^
Inferonasal quadrant	138.49 ± 40.08	144.29 ± 45.55	134.03 ± 34.86	0.093^c^
Inferotemporal quadrant	170.79 ± 52.01	180.92 ± 59.32	162.99 ± 44.33	0.089^c^
*P* value^b^	<0.001^d^	<0.001^d^	<0.001^d^	
Vortex vein's CVI, %				
Superotemporal quadrant	36.63 ± 3.81	37.33 ± 3.74	36.10 ± 3.80	0.023^c^
Superonasal quadrant	34.51 ± 6.27	35.42 ± 6.02	33.81 ± 6.39	0.023^c^
Inferonasal quadrant	28.73 ± 7.21	30.02 ± 6.02	27.74 ± 7.46	0.001^c^
Inferotemporal quadrant	33.85 ± 6.00	35.01 ± 6.29	32.97 ± 6.39	<0.001^c^
*P* value^b^	<0.001^d^	<0.001^d^	<0.001^d^	
Submacular area				
ChT, µm	293.28 ± 101.86	305.17 ± 98.99	284.14 ± 103.51	0.063^c^
CVI, %	41.71 ± 7.64	42.48 ± 8.44	41.12 ± 6.94	0.046^c^

N/A, not available; ChT, choroidal thickness; CVI, choroidal vascularity index.

Values are presented as mean ± SD.

a *P* value for intergroup comparison between male and female subjects.

b *P* value for intergroup comparison among four quadrants.

c Mann–Whitney *U* test.

d Repeated measures analysis of variance.

Subsequently, the 207 eyes were divided into 6 age groups (Fig. 1H; 20–30 = 55 eyes; 30–40 = 38 eyes; 40–50 = 32 eyes; 50–60 = 30 eyes; 60–70 = 33 eyes; and 70–80 = 19 eyes). Line graphs tracing the tendencies of ChT and CVI with age in the 4 VV quadrants and the submacular area were drawn, as shown in Figures 1F and 1G and Supplementary Table S3. A slight decrease in ChT was observed before the age of 50 years, followed by a steep decline after 50 (see Fig. 1F). Meanwhile, the CVI declined steadily from 20 to 80 years of age (see Fig. 1G). Interestingly, the decrease in ChT was steeper in the submacular area than in the VV quadrants.

### Clinical Factors Associated With ChT and CVI in Healthy Eyes

**Figure 2.**
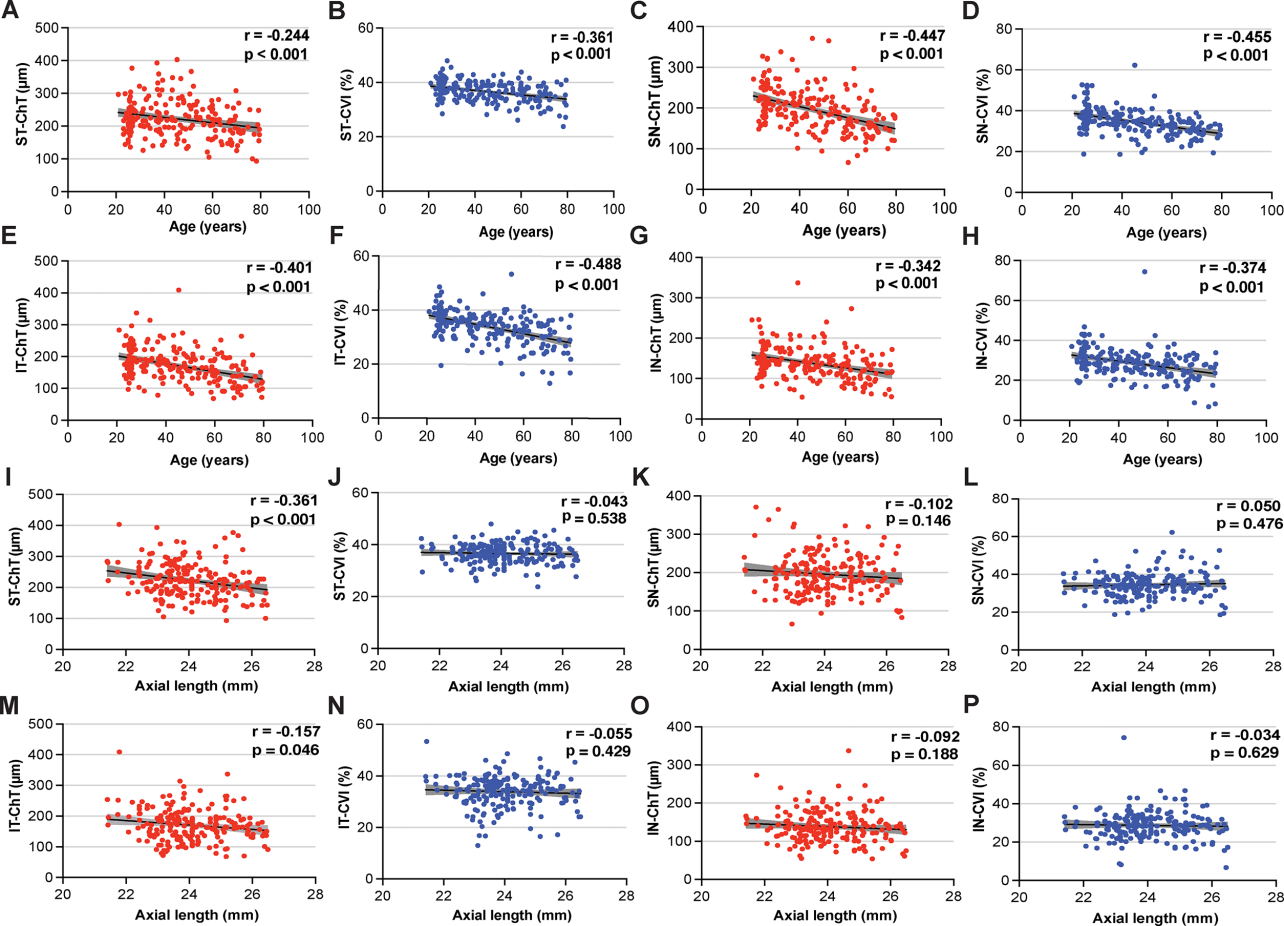
(**A**, **C**, **E**, **G**) Associations between ChT and age in the ST, SN, IT, and IN drainage quadrants, respectively. (**B**, **D**, **F**, **H**) Associations between CVI and age in the ST, SN, IT, and IN drainage quadrants. respectively. (**I**, **K**, **M**, **O**) Associations between ChT and AL in the ST, SN, IT, and IN drainage quadrants, respectively. (**J**, **L**, **N**, **P**) Associations between CVI and AL in the ST, SN, IT, and IN drainage quadrants, respectively. ChT, choroidal thickness; CVI, choroidal vascularity index; ST, superotemporal; SN, superonasal; IT, inferotemporal; IN, inferonasal; AL, axial length.

An analysis of the correlations between the ChT and CVI of each quadrant in terms of age was conducted, the results of which are illustrated in Figure 2. Although ChT exhibited moderate correlations with age in the SN (*r* = −0.447, *P* < 0.001) and IT (*r* = −0.401, *P* < 0.001) quadrants, weak correlations were observed in the ST (*r* = −0.244, *P* < 0.001) and IN (*r* = −0.342, *P* < 0.001) quadrants. Similarly, CVI showed moderate correlations with age in the SN (*r* = −0.455, *P* < 0.001) and IT (*r* = −0.488, *P* < 0.001) quadrants and weak correlations in the ST (*r* = −0.361, *P* < 0.001) and IN (*r* = −0.374, *P* < 0.001) quadrants. Notably, ChT was found to be negatively associated with AL in the ST quadrant (*r* = −0.361, *P <* 0.001) and IT (*r* = −0.157, *P* = 0.046) quadrant, although no such association was detected in the other quadrants. Meanwhile, no correlation was observed between the CVI and AL across all quadrants.

### Characteristics of the Intervortex Venous Anastomosis

**Figure 3.**
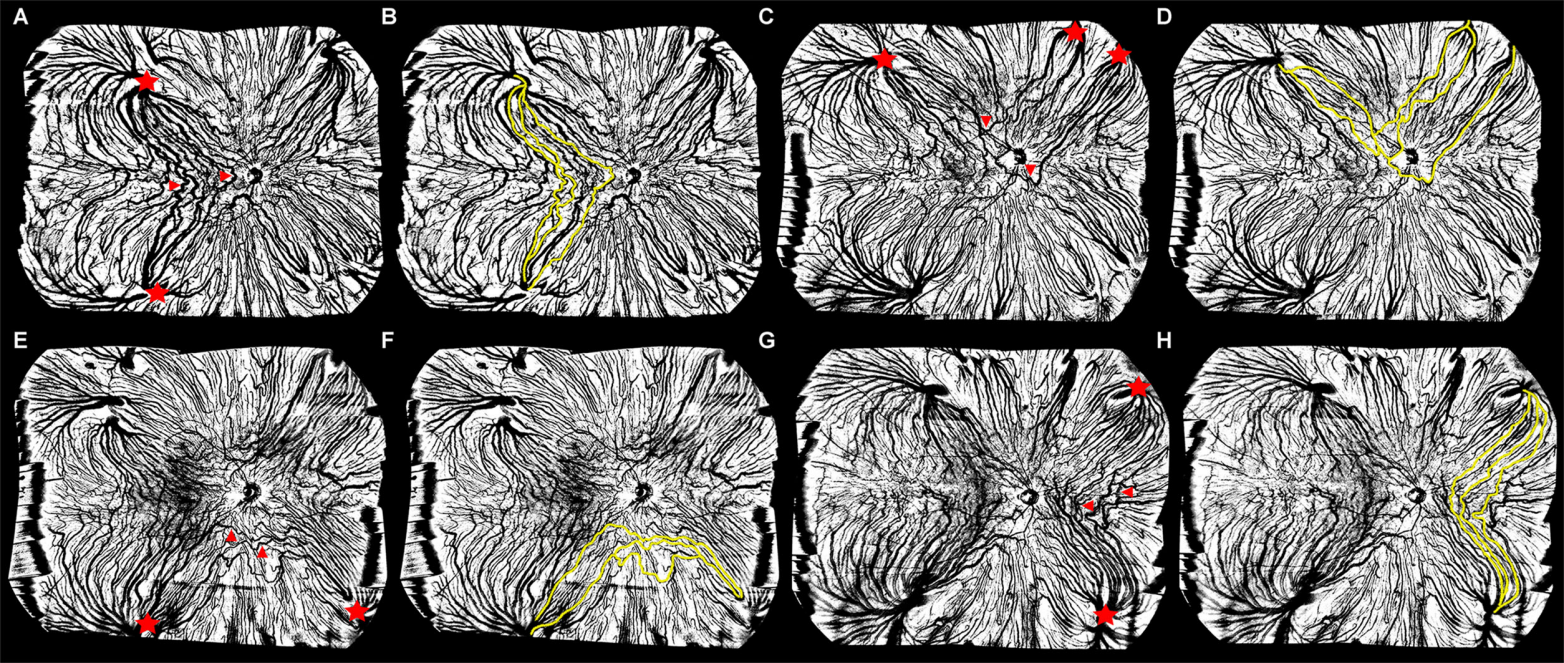
**UWF en face choroidal maps (> 200 degrees).** IVAs in the temporal (**A**), superior (**C**), inferior (**E**), and nasal (**G**) quadrants are *highlighted in yellow* (**B**, **D**, **F**, and **H**). *Red asterisks* indicate the vortex vein (VV) ampullas in adjacent quadrants, whereas the *red arrows* refer to the anastomotic vessels connecting the VV ampullas in adjacent quadrants. UWF, ultra-widefield; IVAs, intervortex venous anastomoses.

The choroidal characteristics related to IVA are presented in Table 3 and Figure 3. As mentioned earlier, in this study, IVAs were classified into temporal (see Figs. 3A, 3B), superior (see Figs. 3C, 3D), inferior (see Figs. 3E, 3F), and nasal (see Figs. 3G, 3H) types. With regard to choroidal characteristics, the occurrence of IVA was observed to be most frequent in the temporal (33.82%) quadrant and least common in the inferior (1.45%) quadrant (see Table 3). Furthermore, the frequency of anastomosis in the macular region was 15.94%. Notably, although no statistical difference between the male and female groups was recorded with regard to the frequency of IVA, the male group exhibited a higher frequency than its counterpart.

**Table 3.** The Characteristics of Intervortex Venous Anastomosis in Healthy Eyes

Characteristics	Total	Male	Female	*P* Value^a^
Eyes, *n*	207	90	117	N/A
Eyes with IVA, *n* (%)	91 (43.96)	45 (50)	46 (39.32)	0.124^c^
Temporal	70 (33.82)	35 (38.89)	35 (29.91)	0.097^c^
Superior	16 (7.73)	12 (13.33)	4 (3.42)	0.008^c^
Nasal	44 (21.26)	23 (25.56)	21 (17.95)	0.103^c^
Inferior	3 (1.45)	2 (2.22)	1 (0.85)	0.414^c^
*P* value^b^	<0.001^c^	<0.001^c^	<0.001^c^	N/A
Macular region	33 (15.94)	17 (18.89)	16 (13.68%)	0.310^c^

N/A, not available; IVA, intervortex venous anastomosis.

Values are presented as number (%).

a *P* value for intergroup comparison between male and female subjects.

b *P* value for intergroup comparison among four quadrants.

c Chi-square test.

## Discussion

In this cross-sectional study, each major choroidal VV drainage system present in the entire post-equatorial fundus in healthy eyes was evaluated using UWF-OCTA. The study results offer three interesting findings related to the choroidal VV drainage system. First, the preferential drainage system in healthy eyes was found to be the ST quadrant, with the VV drainage system adhering to the ST–SN–IT–IN perfusion descending rule. Second, higher blood perfusion in the VV drainage system was observed for men compared to women. Moreover, this perfusion gradually declined with age. Third, almost half of the healthy eyes were observed to have IVA in the choroidal vessel networks.

One of the most significant characterizations of the VV drainage system identified in this study is the ST drainage system emerging as the preferential system in healthy eyes. In this context, Mori et al.^6^ offered ICGA-based evidence that the ST drainage route was preferred in 12 out of 18 healthy eyes. Similarly, in the current study, the ChT and CVI of the four quadrants gradually decreased in the order of ST–SN–IT–IN in most participants. Interestingly, the optic fissure closes along the IN aspect of the optic cup and stalk from the fifth week to the seventh week of the development of a human fetus.^20^^,^^21^ Complete or partial failure of optic fissure closure results in the formation of a coloboma in the IN quadrant.^21^ This suggests that the ST–SN–IT–IN perfusion descending law in the choroidal VV drainage system may be attributed to the development of the vertebrate eye. Our previous study demonstrated that the preferred drainage route in central serous chorioretinopathy might be the ST drainage system.^15^ However, the choroidal VV drainage system in central serous chorioretinopathy does not completely adhere to the ST–SN–IT–IN law of descending perfusion. This indicates that the clinical application of this law necessitates further investigation.

Previous studies have reported that men possess a thicker choroid layer in the submacular area than women.^22^^–^^25^ In our study, the choroid layer in the submacular area, as well as in the VV drainage quadrants, was found to be thicker in men than in women, although this difference was not significant. Furthermore, men attained a higher CVI than women with regard to not only the submacular area, but also the four VV drainage quadrants. To our knowledge, CVI is a more stable biomarker of choroidal vessels than ChT.^26^ Thus, the results of this study support the observation that men experience higher perfusion in their choroidal VV circulation system than women. Physiological differences between men and women may have contributed to this choroidal discrepancy. Another reason for this finding could be hormonal status, which has been proven to influence choroidal circulation—for instance, specific receptors of sex hormones have been found on the choroidal layer.^27^^,^^28^ Moreover, some reports have indicated that androgens activate the vascular endothelial growth factor, in turn promoting the recruitment and proliferation of vascular endothelial cells.^29^ However, studies elaborating the underlying mechanisms are still in progress. Finally, the sex differences on perfusion in the VV drainage system may explain why central serous chorioretinopathy and polypoidal choroidal vasculopathy occur more commonly in men.^12^^,^^30^

Consistent with previous studies, we found that ChT in the submacular area decreases with age.^2^^,^^31^^,^^32^ Our study also found that choroidal perfusion in the VV drainage system declines with age — a finding that may be attributed to sympathetic nervous system dysregulation and vascular impairment.^33^^–^^35^ Notably, one unanticipated result obtained in this research is the dramatic decline in choroidal perfusion after the age of 50 years. Age-related macular degeneration is a significant factor contributing to serious vision loss in adults above 50 years of age.^36^ Notably, previous studies found subfoveal ChT to be negatively related to the severity of nonexudative age-related macular degeneration, as well as the rate of geographic atrophy progression, whereas the vessels located in the subfoveal area were found to be positively associated with perfusion in VVs.^15^^,^^37^ In view of these findings, we hypothesize that the VV drainage system may be closely associated with the development of age-related macular degeneration.

Finally, a classification scheme for IVA, accounting for the entire VV drainage system, was established. In this study, IVA was observed in almost half of the healthy eyes. Notably, Hoshino et al.^38^ detected macular IVA in 44% of healthy eyes. In contrast, the frequency of macular IVA in the current study was 15.94%. A possible explanation for this disparity might be the racial differences between the Japanese and Chinese. Another possible explanation for this discrepancy could be the fact that our study evaluated large venous anastomosis but not small IVA, because the former has a greater impact on choroidal blood circulation. Moreover, we found that IVA was most common in temporal quadrant. The IVA in the temporal quadrant is also a common feature of pachychoroid spectrum disorders, which are characterized by increased choroidal thickness and vessels dilation.^8^ As a result, we hypothesize that temporal IVA, especially macular IVA, may be considered a risk factor indicating a subclinical state of pachychoroid spectrum disorders.

This study has a few limitations. First, the cross-sectional study design did not allow for an investigation into the clinical value of the characterization of the VV drainage system with regard to ocular diseases. To address this issue, longitudinal follow-up studies must be conducted to acquire additional clinical information. Second, only Chinese participants were included in this study. However, significant differences in ocular structure, such as AL and retinal pigment epithelium, have been observed among ethnic and racial groups. Third, although UWF-OCTA technology allows for the three-dimensional reconstruction of the VV drainage system, it cannot measure the real blood flow velocity in the VV. Furthermore, no ICGA test was conducted to assess vessel permeability in healthy subjects. Fourth, due to technical limitations, our team was unable to analyze additional details related to the VV network, such as drainage direction, connectivity, and flux.

In summary, we reported the ST–SN–IT–IN perfusion descending law and the ST drainage system as the most common preferential drainage system in healthy eyes. Furthermore, the influence of age- and sex-related differences on the choroidal VV drainage system of healthy eyes was explored. It was found that AL has little impact on VV's perfusion. Moreover, almost half of the healthy eyes had IVA in the UWF choroidal vessel networks. Finally, the VV drainage system may be considered a promising biomarker for ocular diseases.

## Summary

*What is known about this topic:* The knowledge of the VV drainage system in normal eyes mainly comes from ICGA. However, this invasive method is limited to the small-scale studies and only offers two-dimensional imaging of the choroidal structure. To date, numerous studies have investigated the choroidal layer using UWF-OCTA but the complete VV drainage system in healthy individuals has not been explored.

*What this study adds:* In this large-scale cross-sectional study, we evaluated the complete VV drainage system using UWF-OCTA. We reported the ST–SN–IT–IN perfusion descending law in VV drainage system, and almost half of the healthy eyes had IVA in the UWF choroidal vessel networks.

## Supplementary Material

Supplement 1

Supplement 2

Supplement 3

Supplement 4
