# Supplementary material for: Characterization of Vortex Vein Drainage System in Healthy Individuals Imaged by Ultra-Widefield Optical Coherence Tomography Angiography
Source: Transl Vis Sci Technol. 2024 Sep 18;13(9):19. doi: 10.1167/tvst.13.9.19 (PMC11412622; doi:10.1167/tvst.13.9.19)
Supplement: Supplement 1 [file tvst-13-9-19_s001.pdf]

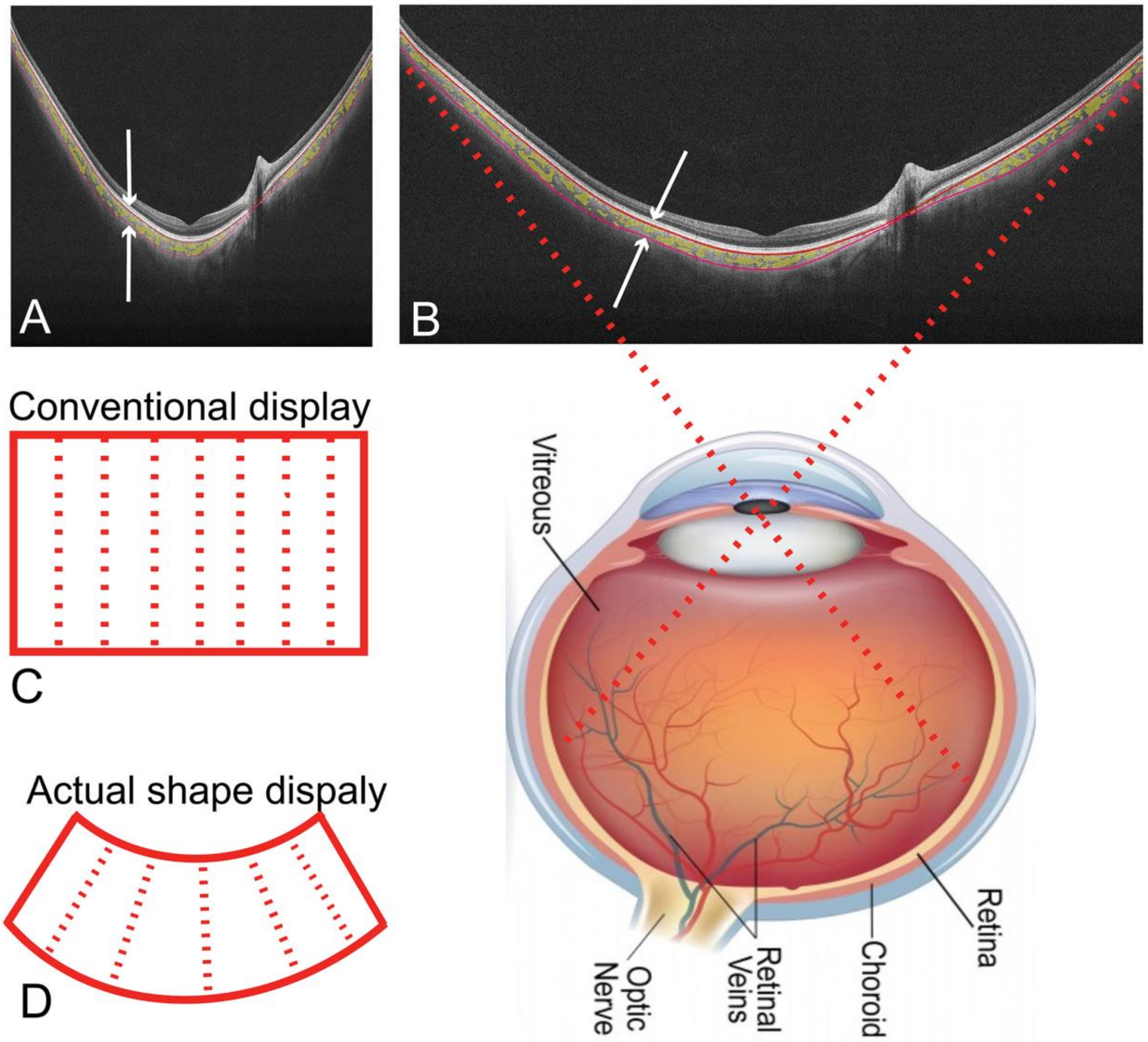

Figure S1. Principle of AL correction in OCT images. (**A**) Conventional image obtained by ultra-widefield OCT and measurement of ChT (white arrows). (**B**) Actual image obtained by ultra-widefield OCT and corrected for AL, and the measurement of actual ChT (white arrows). (**C**) Schematic diagram of the conventional display. (**D**) Schematic diagram of the actual shape display. AL, axial length; OCT, optical coherence tomography; ChT, choroidal thickness.
