# Supplementary material for: Characterization of Vortex Vein Drainage System in Healthy Individuals Imaged by Ultra-Widefield Optical Coherence Tomography Angiography
Source: Transl Vis Sci Technol. 2024 Sep 18;13(9):19. doi: 10.1167/tvst.13.9.19 (PMC11412622; doi:10.1167/tvst.13.9.19)
Supplement: Supplement 3 [file tvst-13-9-19_s003.docx]

**Table S2** Consistency Tests between Investigators A and B for IVA

|  |  | Researcher B | | total |
| --- | --- | --- | --- | --- |
|  |  | IVA | No IVA |  |
| Researcher A | IVA | 120 | 16 | 136 |
|  | No IVA | 12 | 680 | 692 |
| Total |  | 132 | 696 | 828 |

IVA: intervortex venous anastomosis
