# Supplementary material for: Characterization of Vortex Vein Drainage System in Healthy Individuals Imaged by Ultra-Widefield Optical Coherence Tomography Angiography
Source: Transl Vis Sci Technol. 2024 Sep 18;13(9):19. doi: 10.1167/tvst.13.9.19 (PMC11412622; doi:10.1167/tvst.13.9.19)
Supplement: Supplement 4 [file tvst-13-9-19_s004.docx]

**Table S3.** Choroidal Thickness and Choroidal Vascularity Index in Diferent Regions of Various Age Groups

| Age (years) | 20-29 | 30-39 | 40-49 | 50-59 | 60-69 | 70-79 |
| --- | --- | --- | --- | --- | --- | --- |
| ChT (µm) |  | | | | | |
| Submacular area | 309.63 ± 76.70 | 317.61 ± 94.84 | 311.06 ± 101.34 | 320.37 ± 122.02 | 244.41 ± 104.53 | 205.93 ± 73.33 |
| Superotemporal quadrant | 227.49 ± 44.96 | 237.60 ± 63.39 | 230.47 ± 65.41 | 232.60 ± 53.93 | 199.60 ± 43.98 | 178.50 ± 45.09 |
| Superonasal quadrant | 221.22 ± 43.96 | 208.15 ± 48.60 | 201.07 ± 53.42 | 189.57 ± 51.22 | 161.61 ± 49.34 | 156.05 ± 28.11 |
| Inferotemporal quadrant | 190.23 ± 49.08 | 181.89 ± 47.88 | 184.13 ± 56.38 | 160.21 ± 42.52 | 142.41 ± 44.22 | 133.61 ± 47.69 |
| Inferonasal quadrant | 153.86 ± 35.61 | 139.94 ± 32.61 | 141.97 ± 46.99 | 138.30 ± 38.18 | 122.31 ± 41.78 | 112.61 ± 34.56 |
| CVI (%) |  | | | | | |
| Submacular area | 46.17 ± 4.93 | 43.66 ± 6.24 | 42.59 ± 5.00 | 39.85 ± 9.19 | 36.39 ± 7.50 | 35.32 ± 8.23 |
| Superotemporal quadrant | 38.29 ± 3.64 | 37.12 ± 3.11 | 36.45 ± 3.37 | 36.44 ± 3.88 | 35.32 ± 3.37 | 33.48 ± 4.60 |
| Superonasal quadrant | 38.12 ± 6.84 | 35.68 ± 4.46 | 34.14 ± 7.48 | 33.64 ± 4.33 | 30.79 ± 4.46 | 29.80 ± 3.86 |
| Inferotemporal quadrant | 37.36 ± 4.88 | 34.90 ± 3.94 | 34.19 ± 4.23 | 32.86 ± 6.89 | 30.71 ± 5.43 | 27.89 ± 7.28 |
| Inferonasal quadrant | 32.48 ± 6.32 | 28.74 ± 4.92 | 27.77 ± 4.33 | 29.32 ± 10.42 | 25.78 ± 5.78 | 23.57 ± 8.37 |

ChT, choroidal thickness; CVI, choroidal vascularity index.
